# Human migration, diversity and disease association: a convergent role of established and emerging DNA markers

**DOI:** 10.3389/fgene.2013.00155

**Published:** 2013-08-09

**Authors:** Pokhraj Guha, Sanjeev K. Srivastava, Soumen Bhattacharjee, Tapas K. Chaudhuri

**Affiliations:** ^1^Cellular Immunology Laboratory, Department of Zoology, University of North Bengal, SiliguriWest Bengal, India; ^2^Department of Medical genetics, Sanjay Gandhi Post Graduate Institute of Medical Sciences, LucknowUttar Pradesh, India; ^3^Cell and Molecular Biology Laboratory, Department of Zoology, University of North BengalSiliguri, West Bengal, India

**Keywords:** SNP, mitochondrial markers, Y-haplogroup, HLA (MHC), KIR, population diversity, polymorphism

## Abstract

With the gradual development of intelligence, human got curious to know his origin and evolutionary background. Historical statements and anthropological findings were his primary tool for solving the puzzles of his own origin, until came the golden era of molecular markers which took no time to prove it’s excellence in unveiling answers to the questions regarding the migration pattern of human across different geographical regions. As a bonus these markers proved very much beneficial in solving criminal offenses and in understanding the etiology of many dreaded diseases and to design their prevention. In this review, we have aimed to throw light on some of the promising molecular markers which are very much in application now-a-days for not only understanding the evolutionary background and ancient migratory routes of humans but also in the field of forensics and human health.

## INTRODUCTION

Since the origin, spread of mankind across the world has always been an emerging area of interest for modern biologists. Humans migrated “out of Africa” to other geographical locations around the world and eventually diversified into distinct human races populating distinct geographical regions. Human diversity did not only remain restricted to their socio-cultural and linguistic domains but also have penetrated deep inside their genetic root. The wealth of genetic/allelic diversity is not only an excellent resource for human diversity studies but also is highly informative for the study of human genetic predisposition of various diseases (Srivastava et al., 2011; Pál et al., 2012). Thus there is enough reason for growing interests in the field of genetic diversity researches.

Since human genome varies from individual to individual, no two individuals are alike genetically or phenotypically. With the development of various molecular techniques the application of genetics to the study of human evolution gave rise to the fields of molecular evolution and molecular anthropology. Various informative and polymorphic genetic markers were discovered and the gene frequency data emerging out from their analyses largely contributed to the successful study of evolution and diversity of human races worldwide. The use of a good number of uniparental and biparental markers for genetic diversity studies is a recent trend in which Y-haplogroup, mitochondrial DNA (mtDNA), human leukocyte antigen (HLA) and killer-cell immunoglobulin-like receptor (KIR) are the promising ones. The inheritance pattern emerging out from the analyses of these markers stirred a debate on the validity of two distinct models of human dispersals since their inception more than 25 years ago (Stringer and McKie, 1996; Wolpoff and Caspari, 1997).

Multiregional continuity hypothesis (Thorne and Wolpoff, 1992) proposes that humans began to migrate out of Africa about 1.5 million years ago as a single evolving species *Homo sapiens*, distributed throughout the Old World and all regional populations were connected by gene flow as they are today. Some skeletal features developed and persisted for varying periods in the different geographical regions justifying the development of recognizable regional morphologies in the continents of Africa, Europe, and Asia. On the other side, the “recent out of Africa” model (Wilson and Cann, 1992; Stringer and McKie, 1996) proposed that since humans began to radiate out from Africa there have been emergence of several species under the genus *Homo.* This model also argues that *H. sapiens* emerged in Africa approximately 100,000 kilo years ago and began to spread globally, replacing other species of *Homo* that were encountered during its expansion, thereby proposing the development of all current regional morphologies outside Africa, within the last 100,000 kilo years ago. These alternative models of human origin arose from morphological interpretations (Wolpoff and Caspari, 1997). Over the last one and a half decade, molecular evidences from populations of different ethnic regions around the world contributed remarkably to the debate (Hawks et al., 2000).

Herein, we have aimed to throw some light on some of the established and emerging DNA markers which have important implications in studying the population diversity as well as predicting human migration pattern and evolutionary relationships.

## MITOCHONDRIAL DNA

Although mtDNA represents a small fraction of the total genome size of an organism, it has emerged over the last three decades as one of the most popular markers of molecular diversity in animals (Galtier et al., 2009). Human mtDNA is acquired almost exclusively maternally, appears in multiple copies in each cell and possess few important conserved coding sequences thus strengthening the reason for its selection as a marker of choice (Wallace et al., 1999; Galtier et al., 2009).

Human mtDNA is a 16,569 kb circular, double-stranded molecule containing 2 rRNA genes, 22 tRNA genes, and 13 structural genes encoding subunits of the mitochondrial respiratory chain (DiMauro and Schon, 2003). All human mtDNA is inherited maternally because almost always ovum contributes its mitochondria to the developing embryo with only rare exceptions (Giles et al., 1980; Cummins, 2000). In mitochondrial genomes the mutation rate is several times higher than that of nuclear sequences (Brown et al., 1979, 1982; Saccone et al., 2000). As a result of such high rate of mutation events many different mtDNA variants are found in an individual.

**FIGURE 1:**
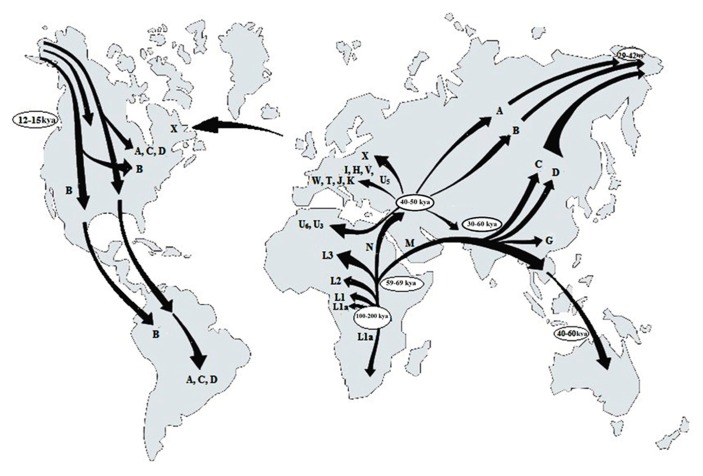
**Geographical distribution of the human mtDNA haplotypes (letters in map) deducing the origin of anatomically modern humans and presumable routes of human migrations “out of Africa” (adopted and modified from Witas and Zawicki, 2004)**.

The application of mtDNA to trace the evolutionary pattern and the migration events in human is based on the fact that certain haplotypes are observed in peoples of certain geographical regions of the World (**Figure 1**) which according to Witas and Zawicki (2004), might have occurred due to accumulation of mutations in different maternal lineages as people migrated and started populating new regions. Cann et al. (1987) has also showed that the highest variation of mtDNA sequences occurs in the African populations. The first human mtDNA lineages described in Africa were L1, L2, and L3, with the L1a subcluster being the oldest (Watson et al., 1997). All of them are still frequent in sub-Saharan Africa, the region having the highest diversity of mtDNA across the world and considered to be the place of origin of all mtDNA sequences (Jorde et al., 1998). Based on mtDNA sequences there are two major migratory routes from Africa (Maca-Meyer et al., 2001). The southern route representing the haplogroup M expansion can be traced from Ethiopia through the Arabian Peninsula to India and Eastern Asia. However, the M haplogroup diversity is greater in India (Kivisild et al., 1999) than in Ethiopia (Quintarna-Murci et al., 1999). The northern route split into three main clusters. The first cluster comprising of the haplogroups W, I and N1b are found in Europe, The Middle East and Caucasia and also in Egypt and Arabian Peninsula. The next group divided into haplogroup X and A, common in Europe and Asia respectively. The third cluster subdivided into four lineages of which the first one gave rise to haplotype B found in Japan, East Asia, and Southern Pacific Archipelago, the second formed haplogroups J and T, whereas H and V, belong to the third cluster, their derivatives being found in Europe, North Africa and Central Asia. The fourth lineage is U and the highest frequencies of its sub-haplogroups are found in India (U2, U7), North Africa (U6, U3) and in Europe (U5; Witas and Zawicki, 2004). According to Richards et al. (1998), the major European mtDNA lineages are U5, H, I, J, K, T, V, W, and X. Haplogroup J encompassing about 16% of European mtDNA content, is probably the only one imported to Europe by the neolithic farmers. Recent studies indicate an early invasion of a single, ancestral lineage of Asian origin in America (Bonatto and Salzano, 1997; Silva et al., 2002). The four most common American haplogroups – A, B, C, and D, although old, have similar nucleotide polymorphism, suggesting their common origin (Silva et al., 2002). The variability pattern of particular mtDNA sequences such as the ATP6 gene from different temperature zones confirm the involvement of selection factors as for, e.g., climate in shaping regional mtDNA variants (Mishmar et al., 2003).

Wallace et al. (1999) suggested that different mtDNA haplotypes may be involved in modulating oxidative phosphorylation, thereby influencing the physiology of individuals predisposing or protecting them from certain diseases. Associations with specific mtDNA haplotypes have been observed for several diseases such as cardiomyopathy (Shin et al., 2000), Alzheimer disease and dementia with Lewy bodies (Chinnery et al., 2000), and multiple sclerosis (Kalman et al., 1999). A recent trend in functional studies of mtDNA mutations has been the use cybrid cells in which established human cell lines are first depleted of their own mtDNAs and then repopulated with various proportions of mutated mtDNA genomes (King and Attardi, 1989). Thus mtDNA haplogroup diversity are eligible enough to answer many of our questions regarding our evolutionary history and also for finding the underlying causes of certain diseases of the human race.

## Y HAPLOGROUP DIVERSITY

**FIGURE 2:**
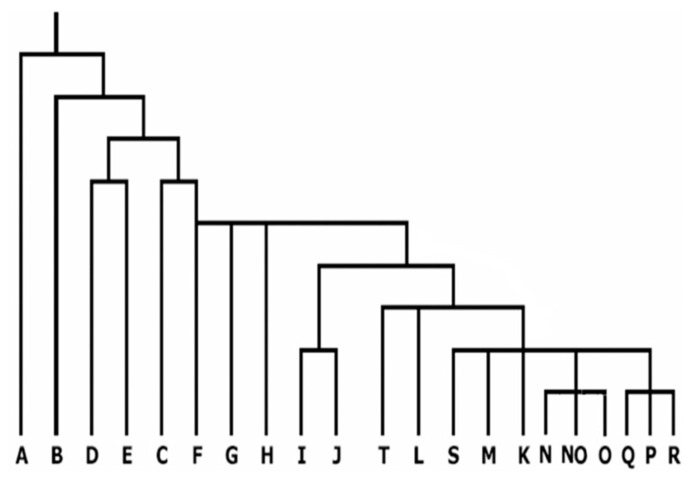
**Current phylogenetic relationships of the 20 major haplogroups of the global Y chromosome gene tree (adopted from Chiaroni et al., 2009)**.

Genetic markers on the non-recombining portion of the Y chromosome have gradually emerged as an important tool for analyzing human phylogenetic relationships. These markers represent human genetic diversity based on single nucleotide polymorphisms (SNPs) on the Y chromosome. There is now extensive knowledge regarding the geographic origins of Y-SNPs based on studies of global populations (Hammer et al., 2001; Jobling and Tyler-Smith, 2003). Because of the high geographic specificity of Y-SNPs (Hammer and Zegura, 1996; Jobling and Tyler-Smith, 2003), SNP haplogroups can be used directly to measure admixture among diverse populations without resorting to more complex models of admixture (Paetkau et al., 1995; Bertorelle and Excoffier, 1998). The present nomenclature system of Y chromosome genotypes has defined 20 main haplogroups, designated A through T (Karafet et al., 2008). The 20 haplogroups are shown in **Figure 2** (adopted from Chiaroni et al., 2009). It is evident that the haplogroups A and B constitute the deepest branches in the phylogeny and are restricted to Africa, thereby strengthening the evidence that modern humans first arose there (Stringer, 2002; Goebel, 2007). The third predominantly African haplogroup E, diversified some time afterward, probably descending from the East African population that generated the “out of Africa” expansion. Haplogroups G–J, T, and L are more prevalent in regions constituting Europe, Middle East, and certain regions of western Asia with extensions to Arabia and India. Haplogroup R is constrained to central and western Asia and to a large portion of Europe. Haplogroup N is frequent across boreal and north-west Asia and O in south-east Asia including the islands and part of New Guinea. Haplogroups C and Q display Asian ancestry and hold the unique privilege of having settled America. Not surprisingly their origin seems to have been in north-east Asia. The absence of haplogroup N in the Americas indicates that its spread across Asia happened after the submergence of the Bering land bridge. It is likely that haplogroup C entered America after Q, even though C originated phylogenetically earlier than Q (Chiaroni et al., 2009).

Y haplogroup diversity has been carried out by Debnath et al. (2011) in the Sub-Himalayan Terai and Dooars population of north-eastern India (Debnath et al., 2011). The study showed that sub-Himalayan paternal gene pool is extremely heterogeneous. Three major haplogroups, namely H, O, and R, are shared across the four linguistic groups that inhabit the area namely, the Tibeto-Burman, Austro-Asiatic, Indo-European and Dravidian.

Earlier studies indicated that Y-chromosome polymorphisms were geographically restricted and that FST values for the NRY were higher than those for mtDNA (Jobling and Tyler-Smith, 1995; Cavalli-Sforza and Minch, 1997; Underhill et al., 1997; Hammer et al., 1998; Perez-Lezaun et al., 1999). Hammer et al. (2001) constructed the hierarchical tree for Y chromosome diversity and showed that the root of the tree occurs between two haplotype sets (H1–H4 and H5–H10) which are entirely restricted to the African continent, thereby supporting the hypothesis of an African origin of contemporary NRY lineages (Hammer et al., 1998; Underhill et al., 2000). In general, the within-populations variance component for Y-chromosome data is much smaller than the values reported for mtDNA (Excoffier et al., 1992; Seielstad et al., 1998; Kittles et al., 1999; Jorde et al., 2000). Hammer et al. (2001) made a major conclusion that global human NRY variation is structured, with a significant amount of intergroup variation partitioned among African, Native American, and Eurasian/Oceanian populations. Based on polymorphism of 43 biallelic markers nested cladistic analysis (NCA) was performed by Hammer et al. (2001), wherein two important findings of the NCA were that Europe was a “receiver” of intercontinental signals primarily from Asia, and there existed a large number of intracontinental signals within Africa. AMOVA analyses of Hammer et al. (2001) also supported the hypothesis that patrilocality effects are evident at local and regional scales, rather than at intercontinental and global levels.

Additionally, Y-chromosome microsatellites find extensive application in forensic researches whereby databases of population haplotype frequencies are established for Europe, the United States and for Asia. Y microsatellite analysis provides assailant specific profile during diagnosis of the rape case when the rapist is mainly azoospermic (Sibille et al., 2002). Paternity testing by Y microsatellite profiling finds application in cases of male child to trace the actual identity of father (Rolf et al., 2001). Thus it can be said that Y chromosome SNPs are very efficient markers for not only evolutionary studies but also for forensic researches.

## HUMAN LEUKOCYTE ANTIGEN

The major histocompatibility complex (MHC)/HLA is unique in that it is the most polymorphic genetic system in the human genome and the only system to display functional polymorphism (Marsh et al., 2000; Spinola et al., 2005). Due to its high polymorphism, tight linkage among the loci and non-random association of alleles this system has become interesting from perspective of population genetics (Bharadwaj et al., 2007; Agrawal et al., 2008). All the regions of HLA are known to be highly polymorphic, constituting several closely linked loci each with large number of genes that can be further split into many allelic types differing only in their nucleotide sequences. Therefore the importance of this system in the study of polymorphism and their significance in population selection and survival and in providing clues to mechanism of generation as well as maintenance of this variability within the populations is immense.

Human leukocyte antigen polymorphism study has been carried out in many of the ethnic populations of India including the primitive tribal group Toto (Debnath and Chaudhuri, 2006; Agrawal et al., 2007). Debnath and Chaudhuri (2006) had shown earlier that HLA-B14 has the highest known frequency in the Toto population of India (32.5%) when compared to that of the other world populations. HLA polymorphism-based affinities of the north and north-eastern populations of India has also been carried out by Agrawal et al. (2008), where they have shown that among the Rajbanshi caste population of India, DRB1 × 1101, DRB1 × 1201, DRB1 × 1501 and DRB1 × 080× were the predominant alleles within the HLA DRB1 locus while DQA1 × 0102, DQA1 × 0201, DQA1 × 0301 and DQA1 × 0501 were the most frequent alleles of the DQA1 locus. At the DQB1 locus, DQB1 × 0201, DQB1 × 0301, DQB1 × 0501, and DQB1 × 0601 were seen to be in high frequency. Among all the three loci haplotypes studied, DRB1 × 0701-DQA1 × 0201-DQB1 × 0501, DRB1 × 1101-DQA1 × 0201-DQB1 × 0301, and DRB1 × 1501-DQA1 × 0601-DQB1 × 0201 are unique in the Rajbanshis. Such HLA profile suggest proximity with the mongoloids (Agrawal et al., 2008).

Apart from being an invaluable tool for population genetic studies, MHC polymorphism has important role in organ transplantation and human disease associations. HLA associations have also helped in defining syndromes of disease categories having common/shared pathogenic mechanism like ankylosing spondylitis and related spondylo-arthropathies that are presumed to be associated with HLA-B27. HLA association studies in infectious and autoimmune diseases show the presence of susceptibility and protective alleles in populations of different ethinic origins (Hill et al., 1991; Carrington et al., 1999; Bowness, 2002). HLA association for psychotic disorders was documented by Debnath et al. (2005, 2006).

Human leukocyte antigen associations with diseases vary in different populations. Disease predisposing genes and their molecular subtypes could help to determine and predict the incidence of the diseases in some populations. It is therefore important to have a population based database of HLA alleles and their frequencies of prevalence in healthy individuals so that disease predisposing influence of a particular phenotype could effectively be assessed in the populations.

Being a functionally a polymorphic system, investigations into the distribution of MHC alleles in world populations are very important in this regard since the MHC genetic makeup of each of these populations would reflect interplay of both the basic genetic origin and effects of natural phenomenon such as founder effect and environmental selection. Differences in the prevalence of HLA alleles in different populations in varied environmental conditions could be utilized to assess the role of each of these alleles in conferring survival advantage to human populations.

## KILLER-CELL IMMUNOGLOBULIN-LIKE RECEPTORS

Killer-cell immunoglobulin-like receptors were first described by Harel-Bellan et al. (1986) and was initially known as “killer inhibitory receptor.” The KIR family of polymorphic and highly homologous genes is located on chromosome 19q13.4, within the 1 Mb leukocyte receptor complex. A total of 17 genes have been identified in the family in agreement with the HUGO Genome Nomenclature Committee (HGNC), of which 15 are functional and 2 are pseudogenes (Marsh et al., 2003). Arrayed in a head-to-tail fashion, KIR genes stretch over a 150 kb domain of DNA with each gene being approximately 10–16 kb in length (Uhrberg et al., 1997). Separation between all loci approximates a 2 kb stretch of DNA with the exception of a 14 kb sequence upstream from 2DS4 (Wilson et al., 2000). The probability of two individuals inheriting the same KIR genotype is slim, with their expression varying clonally, adding yet another layer of complexity. Although KIR haplotypes differ from each other in the number and type of genes (Uhrberg et al., 1997; Witt et al., 1999; Crum et al., 2000; Norman et al., 2001) the genes 2DL4, 3DP1, 3DL2, and 3DL3 are present in virtually all haplotypes and have therefore been termed framework loci (Wilson et al., 2000). Based on KIR gene content two groups of haplotypes (A and B) can be distinguished in human (Uhrberg et al., 1997). Group A KIR haplotypes contain the inhibitory KIR genes along with KIR2DS4 as the only activating receptor. Group B haplotypes contain various combinations of 2DS1, 2DS2, 2DS3, 2DS5, 3DS1, and 2DS4. Group A haplotypes do not vary in gene content, however, extensive variation at the allelic level can be noted (Shilling et al., 2002). In contrast, the group B haplotypes show substantial variations in gene content but only moderate allelic polymorphism can be noticed (Yawata et al., 2002a,b).

Immunogenetic studies based on KIR genes in different ethnic populations around the world show significant differences in the distribution of group A and B haplotypes. Whereas in the Japanese population group A genotypes (i.e., individuals homozygous for two group A haplotypes) were found at frequencies well above 50%, only a single individual out of 67 exhibited a group A genotype in a survey among Australian Aborigines (Toneva et al., 2001; Yawata et al., 2002a,b).

The KIR frequencies of many of the ethnic populations were analyzed worldwide. In one such work, KIR gene profile was studied for the Rajbanshi population, an essential caste population of Sub-himalayan part of north-eastern India (Guha et al., 2013). It was shown that although clustered with the Indian population, KIR gene pool of the Rajbanshis has received a significant Tibeto-Burman influence. This view is also supported by the Y-chromosome haplogroup diversity study which have shown that although totally absent in the Indo-European speaking castes from east India, the O3 haplogroup is considerably shared between Rajbanshis and other such Tibeto-Burman groups (Debnath et al., 2011).

**FIGURE 3:**
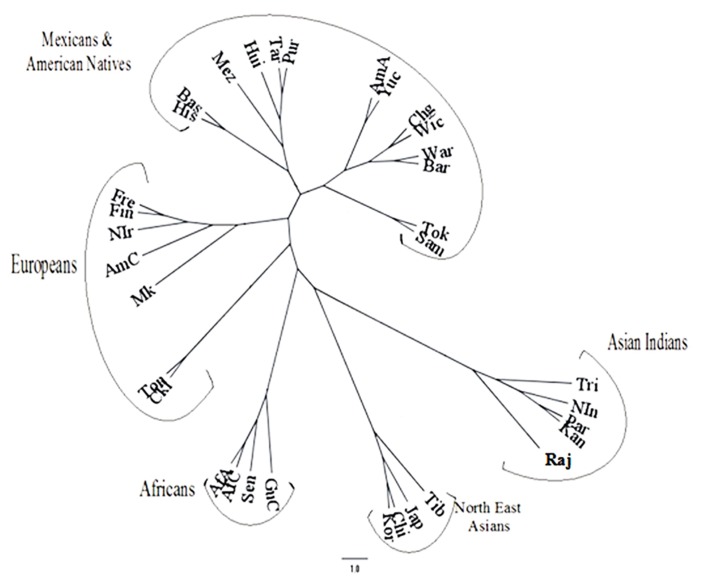
**Phylogenetic dendrogram based on REML analysis of the KIR genotypic profiles in the Rajbanshis and other previously studied World populations (adopted from Guha et al., 2013)**.

The frequencies of the inhibitory KIR genes in most of the world population groups are very high except those on the B haplotypes, i.e., KIR2DL2, KIR2DL5A, and KIR2DL5B. Detailed analysis revealed that indigenous populations such as aborigines and Amerindians have outlying frequencies of the KIR genes. Obviously there is a close inverted correspondence between the frequencies of KIR3DL1 and KIR3DS1 genes in an individual population. Based on KIR haplotype B genes Middleton and Gonzelez (2009) concluded that the population distribution of KIR genes was related to geography like a good anthropological marker such as HLA or Y chromosome. Unlike many other populations (Middleton et al., 2007), the Japanese population showed that the frequency of one allele of each of the KIR genes KIR2DL1, KIR2DL2/2DL3, KIR2DL4, KIR3DL1/S1, KIR3DL2, and KIR2DS4 have higher frequency compared to the next frequent allele (Yawata et al., 2006). This made Parham (2005) to predict a skewed distribution of KIR variants in the Japanese population, reflecting a distinct history of directional and balancing selection. A phylogenetic dendrogram based on KIR genotype frequencies has been shown in **Figure 3** (adopted from Guha et al., 2013) to depict the relation of different World populations.

In humans, KIRs recognize HLA class I proteins leading to the inhibition or activation of cytotoxic cell activity and cytokine production by T and NK cells thus focusing on the role of these receptors in immunological responses of NK cells (Lanier, 1998). The interaction of KIR3DS1 with HLA-B alleles that encode molecules with isoleucine at position 80 (HLA-B Bw4-80Ile) resulted in delayed progression of HIV infection to AIDS (Martin et al., 2002). KIR 2DL4 binds to HLA-G (Ponte et al., 1999; Rajagopalan and Long, 1999), a non-classical class I molecule that is expressed on the human trophoblast, and the resulting receptor-ligand interaction may confer some protection against maternal NK or T cell-mediated rejection of the hemi-allogeneic fetus. These are just a few to mention the roles of KIR-HLA interactions in disease pathogenesis. Moreover, the degree of KIR-HLA interactions may determine the success rate of haematopoietic cell replacement therapy in certain leukemias. Thus this family of receptor on NK cells is turning out to be a hotcake for researchers throughout the World in human evolutionary and disease association studies.

## CONCLUSION

Human have developed their interest in unveiling the mysteries of human migratory pattern and evolutionary trends since his origin. These above mentioned markers are serving the scientific world to trail back through time to understand the dispersal pattern of humans. To add to their importance, these markers are also responsible for understanding the underlying etiology of certain disease pathogenesis. Application of these markers especially Y-SNPs in forensics has been an interesting achievement in the past decade. Apart from these markers, a group of recently emerging markers which are gaining the attention of the researchers all over the world are the toll-like receptors (TLRs; Schwartz and Cook, 2005). In addition to their broad effect on the immunity, they have immense importance in the pathogenesis of human diseases. Further knowledge on the effect of TLR polymorphisms in disease progression may help in the assessment of disease risk and in developing newer therapies accordingly (Schwartz and Cook, 2005). Needless to say further researches and careful investigation is still on demand to decode the full potential of these markers for the benefit of mankind.

## Conflict of Interest Statement

The authors declare that the research was conducted in the absence of any commercial or financial relationships that could be construed as a potential conflict of interest.
